# Targeted Co-Delivery of siRNA and Methotrexate for Tumor Therapy via Mixed Micelles

**DOI:** 10.3390/pharmaceutics11020092

**Published:** 2019-02-21

**Authors:** Fei Hao, Robert J. Lee, Chunmiao Yang, Lihuang Zhong, Yating Sun, Shiyan Dong, Ziyuan Cheng, Lirong Teng, Qingfan Meng, Jiahui Lu, Jing Xie, Lesheng Teng

**Affiliations:** 1 School of Life Sciences, Jilin University, Changchun 130012, China; haofei16@mails.jlu.edu.cn (F.H.); lee.1339@osu.edu (R.J.L.); yangcm425@163.com (C.Y.); zhonglh16@mails.jlu.edu.cn (L.Z.); 18744026372@sina.cn (Y.S.); sydong16@mails.jlu.edu.cn (S.D.); chengzy1316@mails.jlu.edu.cn (Z.C.); tenglirong@jlu.edu.cn (L.T.); mengqf@jlu.edu.cn (Q.M.); lujh@jlu.edu.cn (J.L.); 2 College of Pharmacy, The Ohio State University, Columbus, OH 43210, USA

**Keywords:** drug co-delivery, methotrexate, siRNA, antitumor effect, mixed micelles, targeted delivery system

## Abstract

A combination of chemotherapeutic drugs and siRNA is emerging as a new modality for cancer therapy. A safe and effective carrier platform is needed for combination drug delivery. Here, a functionalized mixed micelle-based delivery system was developed for targeted co-delivery of methotrexate (MTX) and survivin siRNA. Linolenic acid (LA) was separately conjugated to branched polyethlenimine (b-PEI) and methoxy-polyethyleneglycol (mPEG). MTX was then conjugated to LA-modified b-PEI (MTX-bPEI-LA) to form a functionalized polymer-drug conjugate. Functionalized mixed micelles (M-MTX) were obtained by the self-assembly of MTX-bPEI-LA and LA-modified mPEG (mPEG-LA). M-MTX had a narrow particle size distribution and could successfully condense siRNA at an N/P ratio of 16/1. M-MTX/siRNA was selectively taken up by HeLa cells overexpressing the folate receptor (FR) and facilitated the release of the siRNA into the cytoplasm. In vitro, M-MTX/siRNA produced a synergy between MTX and survivin siRNA and markedly suppressed survivin protein expression. In tumor-bearing mice, M-MTX/Cy5-siRNA showed an elevated tumor uptake. In addition, M-MTX/siRNA inhibited tumor growth. Immunohistochemistry and a western blot analysis showed a significant target gene downregulation. In conclusion, M-MTX/siRNA was highly effective as a delivery system and may serve as a model for the targeted co-delivery of therapeutic agents.

## 1. Introduction

The combination of chemotherapy drugs and siRNA is recently emerging as a strategy for cancer therapy [1,2,3]. Rational design for the combination therapy strategy is essential to obtain maximum efficacy with minimum dosage, side effects, and drug tolerance. Methotrexate (MTX) is a dihydrofolate reductase (DHFR) inhibitor and has shown anticancer activity [4,5]. However, MTX monotherapy at high dosage is often associated with systemic toxicity, drug resistance, and low efficacy [6,7]. Therefore, MTX needs to be combined with other agents to reduce side effects and enhance tumor efficacy [8,9]. Small interfering RNA (siRNA) with a length of 20–25 base pairs has drawn much attention from researchers and is regarded as a potential therapeutic modality for cancer [10,11]. A siRNA could specifically silence the expression of the targeted gene through RNA interference [12]. Survivin has recently been found to be a crucial protein to tumor growth and metastasis and is a promising therapeutic target for tumor [13]. Moreover, studies had indicated that the survivin could facilitate tumor drug resistance. The inhibition of survivin expression may boost the chemotherapeutic efficacy of cancer [14]. Thus, a co-delivery of siRNA targeting survivin expression and MTX may be a promising approach to overcome cancer drug resistance [1]. In addition, the distinct mechanisms of MTX and survivin siRNA suggest that their combination may produce synergy [15].

Nanocarriers such as liposomes, micelles, dendrimers, or supramolecular systems have been evaluated as vehicles for the co-delivery of chemotherapeutic drugs and gene agents [2,16]. Micelles for the co-delivery of chemotherapeutic drugs and nucleic acid have been shown to have good stability and to control drug release. They can increase the effectiveness of drug combination therapy and can reduce drug resistance [17,18,19,20]. Compared to the pristine micelles, mixed micelles self-assembled from two or more amphiphilic polymers provide greater flexibility. They have recently drawn much attention for use in combination cancer therapy [21,22]. The mixed micelles are easy to optimize, in terms of kinetic stability, drug loading capacity, size distribution, and the preparation of multifunctional carriers [21,22,23,24]. The mixed micelles also could be prepared with simplified procedures, could achieve a desirable antitumor efficacy, and could reduce variability when expanding to a large scale for clinical application [25,26]. However, the efficacy of the mixed micelles is still restricted due to rapid drug release especially for the co-delivery of two or more different types of therapeutic drugs in blood circulation. Polymer-drug conjugates have been studied as nanomedicine for the improvement of disease treatment efficacy recently [27]. Drug covalently bound to the polymers could avoid the drug dissociation and rapid clearance and could improve the drug stability during dilution or exposure to components in blood. Therefore, a system for the co-delivery of siRNA and MTX was developed based on mixed micelles consisted of functionalized polymer-drug conjugates [28].

**Scheme 1:**
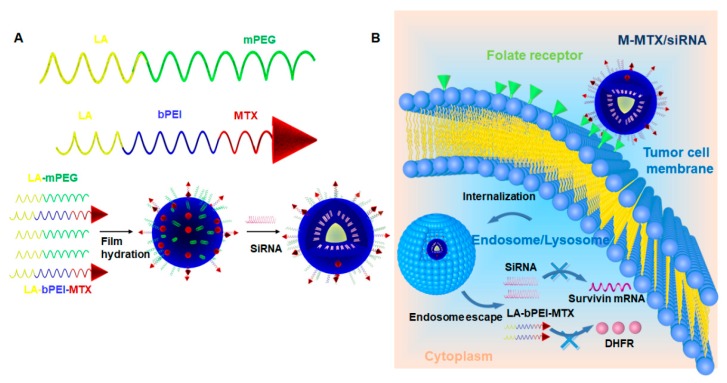
The preparation of the methotrexate (MTX)-conjugated mixed micelles (M-MTX) and the co-delivery of MTX and siRNA to the cytoplasm. (**A**) The amphiphilic polymers of mPEG-LA (linolenic acid-modified methoxy-polyethyleneglycol) and MTX-bPEI-LA (MTX conjugated to linolenic acid-modified branch polyethylenimine) were first synthesized and then self-assembled in one step to form M-MTX and then incubated with siRNA to form the M-MTX/siRNA complexes (M-MTX/siRNA). (**B**) M-MTX/siRNA complexes could be efficiently taken up by the tumor cells through the folate receptor (FR)-mediated endocytosis, could successfully release the loadings to the cytoplasm and could produce a synergy between survivin siRNA and MTX-bPEI-LA with gene silencing and reduced enzyme activity.

MTX conjugated to dendrimers or polymers had previously been shown to retain good antitumor activity in vitro and in vivo [29,30]. Moreover, MTX has been suggested to act both as a targeting ligand and a therapeutic agent in recent studies [31]. When conjugated to dendrimers, MTX may directly target the folic acid (FA) receptor, be internalized into the cell, and then act on its target [32]. Polyethylenimine (PEI) has been used extensively for the delivery of nucleic acids such as siRNA, miRNA, and oligonucleotides because of its superior ability to electrostatically complex with nucleic acids and to facilitate the endosomal escape through its proton sponge effect. However, PEI has had limited clinical use due to its toxicity [33,34]. We previously demonstrated that fatty acid modified PEI showed reduced toxicity and enhanced the efficiency for oligonucleotides delivery [35,36]. Here, MTX was conjugated to linolenic acid modified branched PEI (MTX-bPEI-LA). The MTX-bPEI-LA has several advantages: (i) MTX conjugated to the polymers by amide bond is stable and the MTX conjugated micelles have a long circulation time; (ii) it reduced systemic toxicity compared to free MTX [37]; (iii) bPEI-LA with MTX conjugation may be less toxic than bPEI due to a reduction in charged amino groups [38], and (iv) polymer-MTX conjugates may act in dual roles of a targeting and a therapeutic agent [31,32]. In this study, we also synthesized linolenic acid (LA)-modified methoxy-polyethyleneglycol (mPEG-LA), which was combined with MTX-bPEI-LA to formed mixed micelles (M-MTX) (Scheme 1). In order to evaluate the co-delivery efficiency of the system, we studied their cellular uptake, cytotoxicity, siRNA target modulation, biodistribution and the therapeutic effect in vitro and in vivo.

## 2. Materials and Methods 

### 2.1. Materials 

Branched polyethylenimine (bPEI, 25 kDa) was purchased from Sigma-Aldrich (St. Louis, MO, USA). Methotrexate (MTX) and 3-(4,5-dimethyl-2-thiazolyl)-2,5-diphenyl-2-H-tetrazolium bromide (MTT) were purchased from Shanghai Yuanye Biological Technology (Shanghai, China). mPEG-NH_2_ (2000 Da) was purchased from Yarebio (Shanghai, China). Linolyl chloride (LC) was obtained from Tokyo Chemical Industry Co., Ltd. (Shanghai, China). Survivin siRNA: Sense (5′–3′): mGCAGGUUCCUmUAUCUGUCAdTdT; Antisense (5′–3′): UGAmCAGAmUAAGGAACCUGmCdTdT; Survivin siRNA negative control: Sense (5′–3′): mUUCUCCGAACmGUGUCACGUdTdT; Antisense (5′–3′): ACGmUGACmACGUUCGGAGAmAdTdT, and Cy3, 5′-FAM and Cy5-labeled survivin siRNA for cellular uptake and biodistribution studies were synthesized by Ribo Biochemistry (Guangzhou, China). HeLa cells were purchased from ATCC (Rockefeller, MD, USA). 4′,6-Diamidino-2-phenylindole (DAPI) and Lyso Tracker™ Green DND-99 were purchased from Invitrogen Co. (Carlsbad, CA, USA). The Dihydrofolate Reductase Assay Kit was also purchased from BioVision (S. Milpitas Blvd., Milpitas, CA, USA). All chemical reagents used were of analytical grade.

### 2.2. Synthesis and Characterization of the Amphiphilic Polymers

LA was separately conjugated to the mPEG and b-PEI as shown in Figure S1 using a previously reported method [35,36]. Briefly, linolenic chloride (LC) dissolved in anhydrous dichloromethane (DCM) (Sinopharm Chemical Reagent Co., Ltd., Shanghai, China) was added dropwise to the mPEG2000-NH_2_ and bPEI (25 kDa) anhydrous DCM solution, respectively. After 12 h, the reaction mixture was precipitated and washed three times by diethyl ether (Sinopharm Chemical Reagent Co., Ltd., Shanghai, China). The products mPEG-LA and bPEI-LA were obtained by removing organic solvent in a rotary evaporator (Shanghai Yukang Scientific Instrument Co., Ltd., Shanghai, China) and then vacuumed for 2 h. MTX modified bPEI-LA(MTX-bPEI-LA) was prepared through the reaction of the amino groups of bPEI-LA and carboxy groups of MTX (Figure S1B). The activated reagents of 1-hydroxybenzotriazole (HOBT) (Xiya Chemical Industry Co., Ltd., Linshu, China), *O*-benzotriazole-*N*,*N*,*N′*,*N′*-tetramethyl-uroniumhexafluorophosphate (HBTU) (Xiya Chemical Industry Co., Ltd., Linshu, China), and *N*,*N*-diisopropylethylamine (DIEA) (Sinopharm Chemical Reagent Co., Ltd., Shanghai, China) were first added to the MTX solution to activate the carboxyl groups of MTX for 2 h. Then, the bPEI-LA solution dissolved in anhydrous methanol (Sinopharm Chemical Reagent Co., Ltd., Shanghai, China) was added dropwise to the MTX solution. The mixture was incubated at room temperature for 24 h under nitrogen atmosphere. The reaction mixture was placed in a dialysis bag with a molecular weight cutoff (MWCO) of 8000 to 14,000 Da and dialyzed against deionized water. The dialysate was changed every 4 h. After 48 h, MTX-bPEI-LA was freeze-dried on a Christ epsilon 2-6D LSC (Osterode, Germany). The structures of mPEG-LA, bPEI-LA, and MTX-bPEI-LA were confirmed by ^1^H NMR on a spectrometer from Bruker (Fällanden, Switzerland). mPEG-LA and bPEI-LA were dissolved in deuterated chloroform (CDCl_3_, Cambridge Isotope Laboratories, Inc., Tewksbury, MA, USA). MTX-bPEI-LA was dissolved in deuterated water (D_2_O, (Cambridge Isotope Laboratories, Inc., Tewksbury, MA, USA). The concentration of MTX in MTX-bPEI-LA was determined based on a calibration curve of MTX, and the drug reaction efficiency was calculated. The reaction efficiency of MTX was defined as the ratio of the weight of MTX which was conjugated to the bPEI-LA to the total weight of MTX added to the reaction. The drug loading efficiency was obtained by calculating the ratio of the weight of MTX conjugated to bPEI-LA to the total weight of MTX-bPEI-LA. 

### 2.3. Preparation of MTX-Conjugated Mixed Micelles (M-MTX)

M-MTX was prepared by the self-assembly of MTX-bPEI-LA and mPEG-LA. A specified volume of MTX-bPEI-LA and mPEG-LA solution (with a molar ratio of 1:200) dissolved in chloroform (Sinopharm Chemical Reagent Co., Ltd., Shanghai, China) was mixed together and sonicated for 3 min. Then, the mixture solution was evaporated by a rotary evaporator to remove the chloroform at 37 °C and further under vacuum for 2 h to remove the residual organic solvent and to obtain a film on the flask. To prepare M-MTX, diethyl pyrocarbonate (DEPC)-treated water (Coolaber, Beijing, China) was added to the flask and sonicated for 2 min. The particle size, zeta potential, and polydispersity index (PDI) of M-MTX were measured on a Zeta-sizer Nano ZS90 from Malvern Instruments (Malvern, UK) at 25 °C.

### 2.4. Preparation and Characterization of M-MTX/siRNA Complexes

Gel retardation assays were performed to investigate the ability of M-MTX to complex siRNA using agarose gel electrophoresis. M-MTX with different concentrations and survivin siRNA solutions were first diluted to prepare the M-MTX/siRNA complexes with different N/P ratios. The desired amount of siRNA solution was then mixed with an equal volume of the M-MTX solution by gentle pipetting. The complexes were incubated for 10 min at room temperature before use. Then, 10 μL of the M-MTX/siRNA complexes with different N/P ratios were mixed with 2 μL of 6× loading dye and loaded into a 2% agarose gel. The voltage of electrophoresis (BIO-RAD Laboratories, Hercules, CA, USA) was set up at 100 V and run for 10 min in a Tris-acetate-EDTA (TAE) buffer (Beijing Dingguo Changsheng Biotechnology Co., Ltd., Beijing, China). After that, the gel was placed in a staining solution containing Molecular Probes SYBR® Gold nucleic acid (Invitrogen, Ltd., Willow Creek Road, Eugene, OR, USA) for 30 min. Free siRNA in the complexes could be detected as a band on the gel with a GelDoc-It Ts Imaging System (Analytik Jena US LLC., Upland, CA, USA). The particle size and the zeta potential of the M-MTX/siRNA complexes were measured by Nano ZS90 (Malvern, UK).

### 2.5. In Vitro siRNA Release

The in vitro siRNA release curve of FAM-siRNA loaded M-MTX (M-MTX/FAM-siRNA) in phosphate buffer saline (PBS) was studied. 1 mL M-MTX/FAM-siRNA complexes were transferred into a dialysis bag (MWCO 100 KDa, Shanghai Yuanye Biological Technology, Shanghai, China). The dialysis bag was immersed in 40 mL of PBS and stirred at 37 °C at a speed of 100 rpm. At fixed time intervals, 100 μL of the external solution was withdrawn and replaced with the same volume of fresh PBS. The fluorescence intensity of FAM-siRNA was measured by Bio Tek SYNERGY4 (Winooski, VT, USA) at λ_ex_ = 485 nm and λ_em_ = 535 nm, and the concentrations of FAM-siRNA were measured based on a calibration curve of FAM-labeled siRNA with known concentrations.

### 2.6. Cell Culture

HeLa cells with high folate receptor (FR) expression were used to evaluate the cellular uptake of the M-MTX/siRNA complexes. HeLa cells were cultured in DMEM (Carlsbad, CA, USA) which contained 10% fetal bovine serum (FBS) (Gemini, Woodland, CA, USA) and 1% penicillin-streptomycin (Carlsbad, CA, USA) at 37 °C in a humidified atmosphere of 5% CO_2_.

### 2.7. Hemolytic Analysis of M-MTX and MTX-bPEI-LA on Murine Erythrocytes

Fresh blood samples from healthy mice were collected from the orbital sinus in heparin-coated tubes. Red blood cells (RBCs) were collected by centrifuging at 3000 rpm for 5 min and washed three times with physiological saline solution. Then, the RBCs were dispersed in the physiological saline solution to obtain a 2% erythrocyte standard dispersion (*v/v*). Then, 200 μL various concentrations of MTX and MTX-bPEI-LA (equivalent to MTX-bPEI-LA at concentrations of 0, 40, 80, 100, 150, and 200 μg/mL) were incubated with 1 mL erythrocyte standard dispersion for 3 h. The suspensions were centrifuged, and the absorbance of supernatant (100 µL) was measured at 450 nm. 

### 2.8. The Viability of Cell Cultures Exposed to MTX-bPEI-LA and M-MTX

The carrier cytotoxicity of the MTX-bPEI-LA and M-MTX was studied. The HeLa cells were plated in 96-well microtiter plates (5000 cells per well) and cultured overnight. M-MTX and MTX-bPEI-LA at 3 concentrations (1, 5, and 20 µg/mL) were added. After another 24 h, 20 µL of the MTT solution (5 mg/mL) was added and incubated for 4 h to form formazan crystals. The medium was removed, and the formazan crystals were dissolved by adding 150 µL DMSO and were incubated for 15 min at 37 °C. Absorbance values at 490 nm were measured on Bio Tek SYNERGY4 (Winooski, VT, USA). Relative cell viability was determined and was presented as a viability percentage of the untreated cells.

### 2.9. Cellular Uptake of the M-MTX/Cy3-Labeled siRNA Complexes 

Flow cytometry was first used to investigate the cellular uptake of M-MTX/siRNA complexes. The HeLa cells were seeded in 12-well cell culture plates at a concentration of 1 × 10^5^ per well and cultured for 24 h to be attached to the plates. In order to study the FR targeting ability of the M-MTX/Cy3-labeled siRNA (Cy3-siRNA) complexes, the HeLa cells were first preincubated with free FA (0.1 mM and 1 mM) for 1 h to competitively bind to FR. Mixed micelles without an MTX conjugation but loaded with Cy3-siRNA (M/Cy3-siRNA) were set as a non-FR targeting control. Naked Cy3-siRNA, M/Cy3-siRNA, and M-MTX/Cy3-siRNA with an equivalent siRNA concentration of 100 nM were then added to the plates. After 4 h of incubation, the HeLa cells treated with different formulations were trypsinized, harvested by centrifugation, washed with cold PBS, and resuspended with 4% formaldehyde solution (*w/v*) (Beijing Dingguo Changsheng Biotechnology Co., Ltd., Beijing, China). The fluorescence intensity of the cells was measured on a Beckman Coulter EPICS XL flow cytometer (Brea, CA, USA). The cellular uptake of M-MTX/Cy3-siRNA was further visualized on a confocal laser scanning microscopy (CLSM). The HeLa cells were collected, counted, and then seeded at the bottom of glass flasks for 12 h. The medium was replaced with fresh opti-MEM (Thermo scientific, Rockford, IL, USA) and preincubated with free folic acid (1 mM) for 1 h. The cells were then treated with naked siRNA, M/Cy3-siRNA, and M-MTX/Cy3-siRNA complexes with an equivalent siRNA concentration of 100 nM at 37 °C. After 4 h of incubation, the medium was removed and the cells were washed gently three times with PBS (0.01 M, pH 7.4). Then, the cells were fixed with 4% (*w/v*) formaldehyde for 15 min at room temperature and washed repeatedly with PBS three times to remove the formaldehyde. Subsequently, the nuclei were stained with DAPI for 10 min, and the cells were collected after washing with PBS to remove the residual dye. The uptake of the M-MTX/Cy3-siRNA complexes in the HeLa cells was observed using an LSM710 microscope from Carl Zeiss (Oberkochen, Germany).

### 2.10. Internalization and Endosome Escape of M-MTX/FAM-siRNA Complexes in HeLa Cells 

The HeLa cells were seeded at the bottom of glass flasks at a cell concentration of 1 × 10^5^ and cultured for 24 h. The medium was replaced with fresh opti-MEM and incubated with M-MTX/ FAM-siRNA complexes (100 nM) for 1 h, 2 h, and 4 h, respectively. After washing with PBS, the cells were then incubated with Lyso Tracker™ Red DND-99 for 30 min. Then, the supernatant was removed, and the cells were gently washed. The cells were then sequentially fixed with 4% (*w/v*) formaldehyde and stained with DAPI. After washing away the residual dye, the internalization and endosome escape of M-MTX/ FAM-siRNA complexes was observed on CLSM.

### 2.11. Cell Cytotoxicity of the M-MTX/Survivin-siRNA

The cytotoxicity of the M-MTX/survivin-siRNA complexes was investigated. The HeLa cells were plated in 96-well microtiter plates (1 × 10^4^ cells per well) and cultured overnight. The cells of the designed wells were pretreated with free FA (1 mM) for 1 h. MTX, M-MTX, M-MTX/survivin siRNA negative control, M, M/survivin-siRNA, and M-MTX/survivin-siRNA were added to the wells (survivin siRNA 50 nM, MTX 0.242 μg/mL). M-MTX loaded with a survivin siRNA negative control was defined as an M-MTX/siRNA negative control. After being cultured for 48 h, 20 μL of the MTT solution (5 mg/mL) was added and incubated for 4 h to form formazan crystals. The medium was removed, and the formazan crystals were dissolved by adding 150 μL dimethyl sulfoxide (DMSO, Sinopharm Chemical Reagent Co., Ltd., Shanghai, China). The absorbance values at 490 nm were measured on Bio Tek SYNERGY4 (Winooski, VT, USA). The relative cell viabilities were presented as a viability percentage of the cells treated with different formulations compared to the untreated cell samples which the viabilities were referred to be 100%.

### 2.12. Western Blot Test

The survivin expression was analyzed by western blot. The HeLa cells were seeded in 6-well cell culture plates at a concentration of 1.5 × 10^5^ cells per well for 24 h at 37 °C in a 5% CO_2_ humidified atmosphere. Free FA (1 mM) was preincubated with the cells in the desired wells. MTX, M-MTX, M-MTX/siRNA, M-MTX/siRNA negative control, and M, M/siRNA with an equivalent siRNA concentration to 50 nM were then added to the wells. After 4 h of incubation, the medium was replaced with a fresh medium. After 48 h, the cells of different groups were collected, lysed in radio immunoprecipitation assay (RIPA) lysis buffer containing 1% protease inhibitor cocktail (Sigma-Aldrich, St. Louis, MO, USA) and 2% phenylmethanesulfonyl fluoride (Sigma-Aldrich, St. Louis, MO, USA) and plated on ice for 15 min. Protein fractions were collected by centrifugation at 10,000 rpm at 4 °C for 10 min, quantified by a bicinchoninic acid (BCA) Protein Assay Kit (Thermo scientific, Rockford, IL, USA), subjected to polyacrylamide gel electrophoresis containing 10% sodium dodecyl sulfate (SDS-PAGE), and then transferred to polyvinylidene fluoride (PVDF) membranes (0.45 m, Merck Millipore, Billerica, MA). The membranes were blocked with a 5% bovine serum albumin (BSA) (Sigma-Aldrich, St. Louis, MO, USA) solution (*w/v*) for 4 h at room temperature and incubated with survivin rabbit mAb (71G4B7E, Cell Signaling Technology Inc, Danvers, MA, USA) and GAPDH (ab181602, Abcam, cambridgeshire, UK) antibodies at 4 °C overnight, respectively. Horseradish peroxidase (HPR)-conjugated secondary antibody (Beijing Dingguo Changsheng Biotechnology Co., Ltd., Beijing, China) was added and incubated with the membranes at 4 °C for 4 h. The corresponding protein expression was measured by an electrochemiluminescence (ECL) detection kit (Merck Millipore, Billerica, MA, USA) and visualized by an imaging system (BioSpectrum 600, Analytik Jena US LLC., Upland, CA, USA).

### 2.13. Dose-Dependent Inhibition Efficiency of MTX and M-MTX on Dihydrofolate Reductase (DHFR) Activity 

The inhibition efficiency of MTX and M-MTX on DHFR activity was carried out in accordance to the protocol of the Dihydrofolate Reductase Activity Kit (Colorimetric) from BioVision (S. Milpitas Blvd., Milpitas, CA, USA). Briefly, 40 μL of NADPH (500 μM), 60 μL of the DHFR substrate (15-fold dilution), and 50 μL of a series of concentrations of MTX or MTX-bPEI-LA (an equivalent MTX concentration 62.5 nM to 1000 nM) were added to each well of a 96-well clear plate. Finally, 50 μL of DHFR (250-fold dilution) was added to the wells to initiate the reaction with a total volume of 200 μL per well. The absorbance values at 340 nm were measured for 10 min at room temperature. The wells without MTX and MTX-bPEI-LA were set as the positive controls, and the wells without the DHFR enzyme were set as the negative controls. The Inhibition efficiency was defined as follows:

$$\mathrm{Inhibition}\;\mathrm{efficiency}\;\left(\%\right)=\frac{\left(\Delta OD\;positive\;control-\Delta OD\;sample\right)}{\left(\Delta OD\;positive\;control-\Delta OD\;negative\;control\right)}$$

### 2.14. Establishment of Tumor Model 

The animal experimental protocol was in compliance with the institutional guidelines and was approved by the Experimental Animal Ethics Committee of the School of Life Sciences, Jilin University. The number for the permit for the animal experiment was 201805003 from the Experimental Animal Ethics Committee of the School of Life Sciences, Jilin University. BALB/c nude mice (female, 6–8 weeks) were obtained from Beijing Vital River Laboratory Animal Technology Co., Ltd. (Beijing, China). Tumor-bearing mice were established through the subcutaneous injection of 5 × 10^6^ HeLa cells into the right rear leg of nude mice after the mice were adapted to the new environment for a week.

### 2.15. Accumulation of M-MTX/Cy5-Labeled siRNA (Cy5-siRNA) Complexes in Tumor Tissue

Tumor-bearing mice were injected with Cy5-siRNA, M/Cy5-siRNA, and M-MTX/Cy5-siRNA via the tail vein with an equivalent amount of siRNA (1 nmol). The biodistribution of the complexes was visualized by an IVIS® spectrum system from Caliper Life Sciences (Hopkinton, MA, USA) at the second, fourth, and sixth hour after administration. The mice were anesthetized by administrating a 1% (*w/v*) pentobarbital sodium solution to the abdomen, and the optimized parameter (excitation, 640 nm; emission, 680 nm) was set up for image acquisition at various time points. At the sixth hour after administration, the internal organs (Heart, Liver, Spleen, Lung, and Kidney) and tumors were dissected and then visualized by the In Vivo Imaging System.

### 2.16. In Vivo Antitumor Efficacy of M-MTX/Survivin siRNA Complexes

When the average tumor volume of nude mice was grown to approximately 100–150 mm^3^ (Day 0), the animals were randomized into four groups, each group containing 5 nude mice. Tumor-bearing mice were then injected with saline, free MTX, the M-MTX/siRNA negative control, and M-MTX/siRNA via the tail vein, respectively. The formulations (MTX 500 μg/kg, siRNA 2 nmol) were administered every 3 days. Simultaneously, the volume of the tumor and body weight were measured every 4 days using a vernier caliper and scale. On day 24, the mice were anesthetized with sodium pentobarbital and sacrificed. The tumors were dissected, weighed, and fixed for hematoxylin and eosin (H&E) and immunohistochemistry staining. The expression of survivin in tumor tissue was also analyzed by a western blot assay. The pixel density of the survivin bands compared to the GAPDH bands was quantified for each sample using Image J software (National Institutes of Health, Bethesda, MD, USA).

### 2.17. Histopathologic Analysis

All mice were euthanized with a 1% pentobarbital sodium solution on day 24. Vital organs (Heart, liver, Spleen, Lung, and Kidney) and tumors were dissected in each group and fixed with 4% paraformaldehyde for histopathologic analysis. Tissue sections were cut into 5 microns thick and stained with H&E. For the immunohistochemistry analysis, the tumor tissue sections were first incubated with survivin rabbit mAb.

### 2.18. Statistical Analysis 

Data were expressed as mean ± SEM and graphed by Origin 8.0 (OriginLab Corp., Northampton, MA, USA). The statistical analysis of two group differences and correlations was determined using Student’s *t*-test. * *p* < 0.05 was considered statistically significant. ** *p* < 0.01 and *** *p* < 0.001 were considered highly significant.

## 3. Results

### 3.1. Synthesis and Characterization of MTX-bPEI-LA and mPEG-LA

The chemical structure characterization of mPEG-LA, bPEI-LA, and MTX-bPEI-LA was confirmed by ^1^H NMR (Figures S2–S4). The peak assignment and peak integration were marked with character. In Figure S2, the new characteristic peak of mPEG-LA was found at 3.37 ppm for 1α-NC=O–C (C). The peak assignment of the 1α-NC=O–C indicated that the LC was successfully conjugated to mPEG-NH_2_. Respectively, the characteristic peaks for 1-ethylene, methyl, and methylene of LA were seen at 5.34 ppm (A), 0.88 ppm (F), and 1.62 ppm (E). The characteristic peaks for mPEG were marked with B at 3.65 ppm for ethyl (–H–CH–CH–H). The characteristic peak for amide near 1α-C(=0)–N and 2α-C=C of mPEG-LA was at 2.04 ppm (D). The purity of the mPEG-LA was then calculated to be 91% based on the peak integration of C (1α-NC=O–C, δ = 3.37ppm, 1.01) and F (–H–CH_2_–CH–H, δ = 0.88 ppm, 1.67). As shown in Figure S3, the characteristic peak assignments and peak integrations of PEI and LA were both studied. The characteristic peaks of PEI were marked with C (about 2.3–3.00 ppm, 14.27), and the characteristic peak of LA was marked with A (δ = 5.34 ppm, 0.93) for 1-ethylene, E (1β-C–(C=O)–N, δ = 1.56 ppm, 0.40), and F (–H–CH_2_–CH–H, δ = 0.88 ppm, 0.81). A new characteristic peak was found in B (1α-N-(C)-C and 1-N–C, δ = 3.63 ppm, 1.14) indicating a successful synthesis of bPEI-LA (Figure S3). The purity of bPEI-LA was calculated to be 94.7% based on the peak integration of B and F (Figure S3). Then, the degree of functionalization of bPEI with LA moieties was calculated to 12. The ^1^H NMR spectra of MTX-bPEI-LA conjugates contained not only the characteristic peaks of bPEI (G, δ = 2.89 ppm) and LC (D, δ = 5.32 ppm; F, δ = 0.90 ppm) but also the characteristic signals for 1-benzene (B, δ = 7.71 ppm; C, δ = 7.34 ppm) and 2-pyrazine (A, δ = 8.47 ppm) of MTX (Figure S4). Furthermore, the new characteristic peak at 3.52 ppm (E) due to the generation of an amide bond between bPEI and MTX was also characterized. The results indicated that the MTX was successfully conjugated to the bPEI-LA-NH_2_. The purity of MTX-bPEI-LA was calculated to be 95.6% based on the characteristic peak integration of B (2.08), C (2.08), and E (4.50). The absorption curve peak of the MTX-bPEI-LA was measured at 303 nm. The MTX reaction efficiency and the drug loading efficiency were calculated to be 59.6% and 12.1% respectively through the standard curve of MTX with a known concentration measured by a UV spectrophotometer. The degree of functionalization of bPEI-LA with MTX moieties was also calculated to 15.

### 3.2. Preparation and Characterization of M-MTX and M-MTX/siRNA Complexes

**Figure 1:**
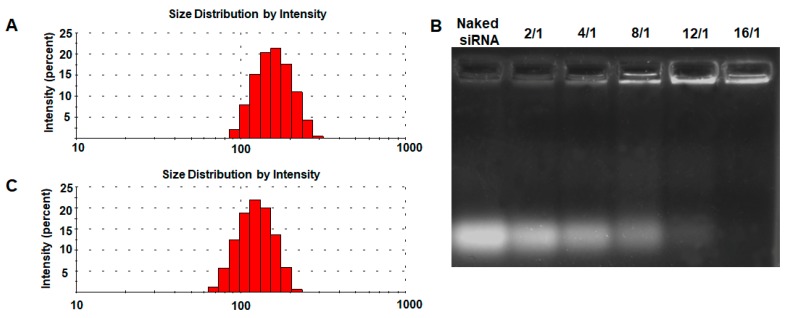
The characterization of the M-MTX and M-MTX /siRNA complexes. (**A**) The particle size of M-MTX was measured by Zeta-sizer Nano ZS90. (**B**) SiRNA was then condensed by M-MTX with a different N/P ratio through the electrostatic interaction. (**C**) The particle size of the M-MTX/siRNA complexes at an N/P ratio of 16/1 was further determined.

M-MTX was prepared by self-assembly of MTX-bPEI-LA and mPEG-LA and had a narrow particle size of 141.8 ± 2.4 nm (PDI = 0.212 ± 0.012) (Figure 1A). SiRNA was then complexed by M-MTX with different N/P ratios (Figure 1B). The result showed that siRNA could be successfully condensed via electrostatic adsorption at an N/P ratio of 16/1 (Figure 1B). M-MTX/siRNA complexes also had a narrow particle size of 124.7 ± 9.1 nm (PDI = 0.231 ± 0.011) (Figure 1C). SiRNA adsorption to the cationic polymer of the carrier may contribute to a decrease in particle size and zeta potential (12.23 ± 0.47 mv decreased to 1.63 ± 0.14 mv). The MTX and the survivin siRNA drug loading efficiencies in the M-MTX/siRNA complexes were then calculated to be 0.17% and 2% (*w/w*) respectively with an N/P ratio of 16/1.

### 3.3. In Vitro FAM-siRNA Release

The in vitro siRNA release curve of FAM-siRNA loaded in M-MTX (M-MTX/FAM-siRNA) was shown in Figure S5. The cumulative release percentage of FAM-siRNA loaded in the M-MTX/siRNA complexes was less than 30% at 48 h. A strong interaction of siRNA with the cationic carrier may play an important role in the slow release of FAM-siRNA loaded in M-MTX. The results showed a controlled release of siRNA and high stability of the M-MTX/FAM-siRNA complexes in PBS (pH = 7.4). The release curve of free MTX in M-MTX in a dialysis bag with an MWCO of 100 KDa was also investigated in PBS (pH = 7.4). However, no free MTX was detected within 72 h. Since MTX was conjugated to the polymers (MTX-bPEI-LA) with a stable amide bond, MTX was hardly released in the release medium at pH = 7.4. The results were consistent with those of the previous study [39].

### 3.4. Carrier Toxicity of M-MTX and MTX-bPEI-LA

PEG has widely been applied to reduce the cytotoxicity, to prolong the circulation time in the blood, and to avoid uptake by the mononuclear phagocyte system of the cationic carriers due to its well-known good biocompatibility [23,24,40]. In order to investigate whether mPEG-LA had a good ability to reduce the toxicity of the mixed micelles, we performed a hemolytic activity assay and cytotoxicity analysis of M-MTX and MTX-bPEI-LA, as shown in Figures S6 and S7. As shown in Figure S6, MTX-bPEI-LA had a much stronger hemolysis effect when the concentrations of MTX-bPEI-LA were greater than 50 µg/mL compared with M-MTX at the same MTX-bPEI-LA concentration. In Figure S7, MTX-bPEI-LA and M-MTX had the same cytotoxicity when the concentration of MTX-bPEI-LA was low (1 μg/mL). When the concentration of MTX-bPEI-LA was 5 μg/mL, MTX-bPEI-LA showed significant carrier toxicity while M-MTX also had low cytotoxicity. When the concentration was 20 μg/mL, both MTX-bPEI-LA and M-MTX showed higher carrier toxicity. These results indicated that the mPEG-LA could reduce the toxicity of the cationic polymer (MTX-bPEI-LA) within a moderate concentration range which may interact with the cell membrane as the previous study suggested [23,24,40]. Thus, the mixed micelles were expected to have a greater potential in the clinical application compared with MTX-bPEI-LA.

### 3.5. Cellular Uptake of M-MTX/Cy3-siRNA in FR-Overexpressing HeLa Cells

**Figure 2:**
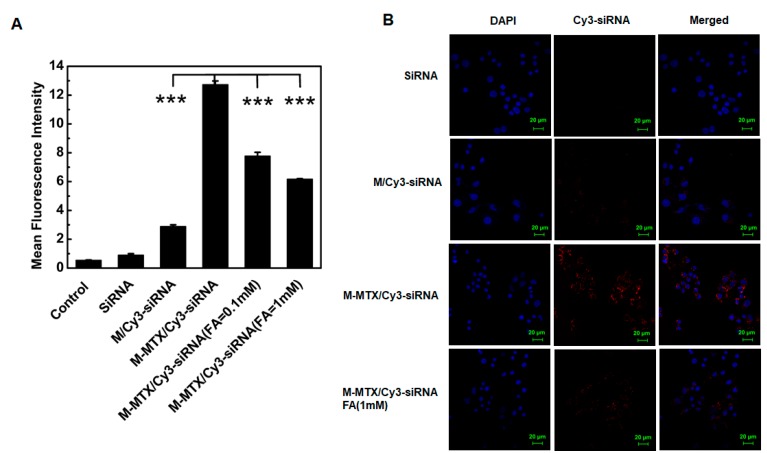
The cell uptake of M-MTX/Cy3-siRNA in the FR-expressing HeLa cells. (**A**) Flow cytometry was used to quantify the cellular uptake of different formulations. The HeLa cells were incubated with M-MTX/Cy3-siRNA, M/Cy3-siRNA, and Cy3-siRNA for 4 h at 37 °C. Two concentrations of free folic acid (FA) (0.1 mM and 1 mM) was added 1 h before the M-MTX/Cy3-siRNA (100 nM) was incubated. *** <. (**B**) The confocal micrographs of HeLa cells were also obtained after incubation with M-MTX/Cy3-siRNA, M/Cy3-siRNA, and Cy3-siRNA for 4 h at 37 °C. Free FA (1 mM) was also added 1 h before the M-MTX/Cy3-siRNA (100 nM) addition. The green bar in the image was 20 μm. The values were the mean ± SEM (n = 3).

The cellular uptake of M-MTX/Cy3-siRNA was investigated in the FR-overexpressing HeLa cells. Flow cytometry results showed that the M-MTX/Cy3-siRNA-treated groups had the highest mean fluorescence intensity and had significant improvement compared with the Cy3-siRNA- and M/Cy3-siRNA-treated groups (*** *p* < 0.001) (Figure 2A). Moreover, the fluorescence intensity of the M-MTX/Cy3-siRNA-treated group could be greatly reduced by adding a free FA (0.1 mM and 1 mM) to cells in advance. The similar results were further visualized by CLSM. The red signal in the cytoplasm treated with M-MTX/Cy3-siRNA was markedly stronger than that of the naked Cy3-siRNA and M/Cy3-siRNA and was also greatly reduced due to the free FA (1 mM) preincubated with HeLa cells (Figure 2B).

### 3.6. Internalization and Endosome Escape of M-MTX/ FAM-siRNA in FR-Overexpressing HeLa Cell

**Figure 3:**
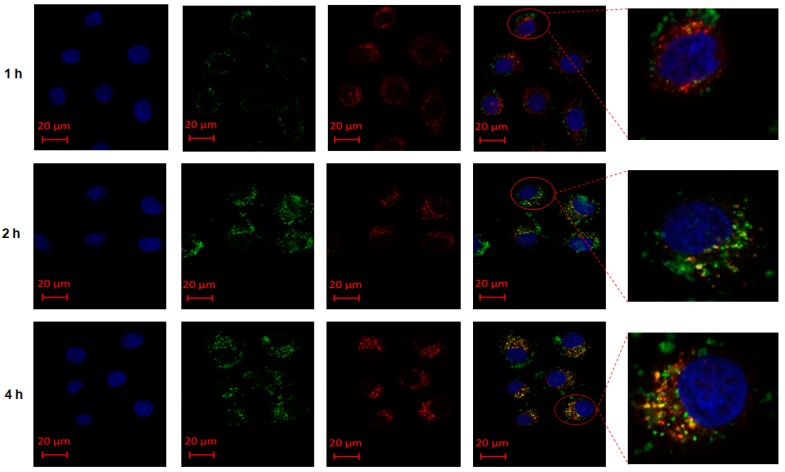
The internalization and endosome escape of M-MTX/siRNA in folate receptor (FR)-overexpressing HeLa cells: The internalization and endosome (red) escape of M-MTX loaded with FAM-siRNA (green) in the Hela cells was visualized by the confocal laser scanning microscopy (CLSM) at the first, second, and fourth hours. The red bar in the images was 20 μm.

The M-MTX/FAM-siRNA complexes could be specifically taken up by HeLa cells. However, the process of internalization, cytoplasmic delivery, subcellular localization, and drug release of FAM-siRNA still remained unclear. Thus, we applied another fluorescent reagent, Lyso Tracker™ Red DND-99, to label the endosomes and to investigate whether FAM-siRNA could escape from the endosomes. As shown in Figure 3, M-MTX/ FAM-siRNA (green signal) started to be internalized to the cells, and the endosomes (red signal) were also found near the cell membrane at the first hour. At the second hour, the overlap of green and red signals gradually increased and the overlapping orange signal started to transport into the cytoplasm. At the fourth hour, the fluorescence signal was completely located in the cytoplasm and a rich green signal was observed to clearly separate from the red signal, indicating that FAM-siRNA were successfully released into the cytosol. The results demonstrated the endocytic process of M-MTX/FAM-siRNA, which finally achieved drug release in the HeLa cells. The proton sponge effect of the cationic polymer of bPEI may play the most important role in achieving endosome escape and drug release [34]. Although some of the amines of bPEI had been reacted with LA and MTX, M-MTX still had a proton sponge effect and achieved siRNA endosomal escape.

### 3.7. In Vitro Biological Activities of M-MTX/Survivin-siRNA

**Figure 4:**
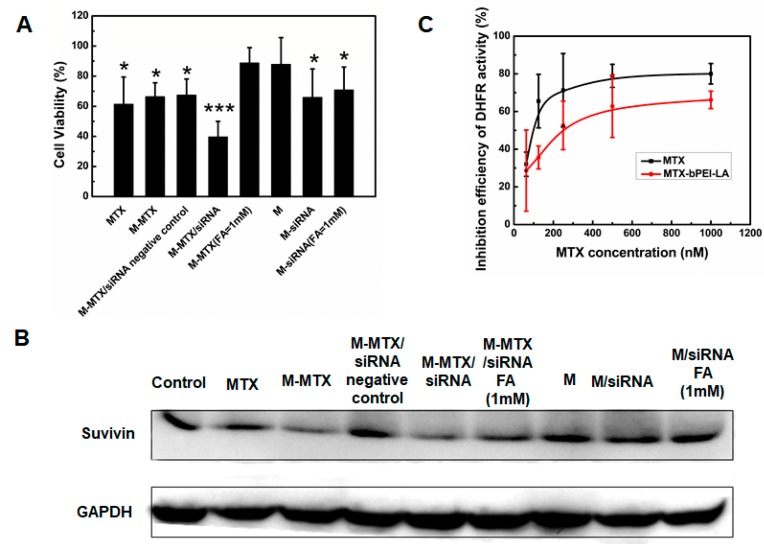
The cytotoxicity, protein expression, and dihydrofolate reductase (DHFR) inhibition activity of M-MTX/siRNA. (**A**) The cell viability was obtained by an MTT assay after incubation with MTX, M-MTX, M-MTX/siRNA negative control, M-MTX/siRNA, M, and M/siRNA for 48 h. * < 0.1, *** < 0.001.(**B**) Survivin expression was further measured by a western blot assay. The competitive inhibition was also investigated by preincubated with FA (1 mM) in advance. (**C**) The inhibition activity of MTX-bPEI-LA to DHFR was performed to evaluate the difference of inhibition activity of the conjugated MTX compared to free MTX.

The synergy of MTX and survivin-siRNA was first studied in HeLa cells by an MTT assay. M-MTX/survivin-siRNA showed enhanced cell cytotoxicity (*** *p* < 0.001) compared to MTX and siRNA alone in vitro (Figure 4A). Simultaneously, the cytotoxic efficiency of the M-MTX/survivin-siRNA-treated group was reversed by free FA(1 mM) while no significant cytotoxicity difference was found between the M/siRNA-treated group preincubated with free FA (1 mM) and the M/siRNA-treated group without FA (1 mM) addition (Figure 4A). The western blot results were also consistent with the cytotoxicity experiments (Figure 4B). M-MTX/survivin-siRNA had the lowest protein expression and could be reversed by the addition of FA (1 mM). MTX was conjugated to bPEI-LA through an amide bond which had previously been demonstrated to be a stable chemical bond and was difficult to degrade. Thus, we assumed that the inhibition of cell viability and protein expression was achieved by MTX-bPEI-LA. To verify this, we investigated the inhibitory activity of MTX and MTX-bPEI-LA on DHFR. As shown in Figure 4C, MTX-bPEI-LA with a larger structure also had a good inhibitory efficiency against DHFR, although slightly lower than MTX, and exhibited a concentration-dependent inhibitory effect. The large structural steric hindrance of MTX-bPEI-LA may result in the reduced binding ability to DHFR. Interestingly, M-MTX had a higher protein expression inhibitory effect in the western blot assay after 48 h compared with free MTX although MTX-bPEI-LA was shown to have a lower inhibitory activity against DHFR (Figure 4B). Survivin expression is closely related to the efficient delivery of survivin siRNA and MTX to the targeted site. This could be explained by a much higher uptake of M-MTX, which had a positive charge and was able to interact with the negatively charged cell membranes. Free MTX, M-MTX, M-MTX/siRNA negative control, and M-MTX/siRNA complexes were incubated with cells only for 4 h and replaced with fresh medium in the western blot assay. M-MTX had a significant decrease in survivin expression due to more M-MTX uptake compared to MTX and the M-MTX/siRNA negative control. The greatest cytotoxicity and protein inhibition caused by M-MTX/siRNA may depend primarily on the enhanced tumor cell uptake of MTX conjugates, while M-siRNA without MTX conjugates with a much smaller amount of cellular uptake showed lower cytotoxicity and more survivin expression. Thus, we proposed that M-MTX had enhanced the anticancer therapy compared to the free MTX and could serve as both a targeting agent and a chemotherapeutic agent for tumor therapy [31,32,41,42].

### 3.8. Biodistribution of M-MTX/Cy5-siRNA in Tumor-Bearing Mice 

**Figure 5:**
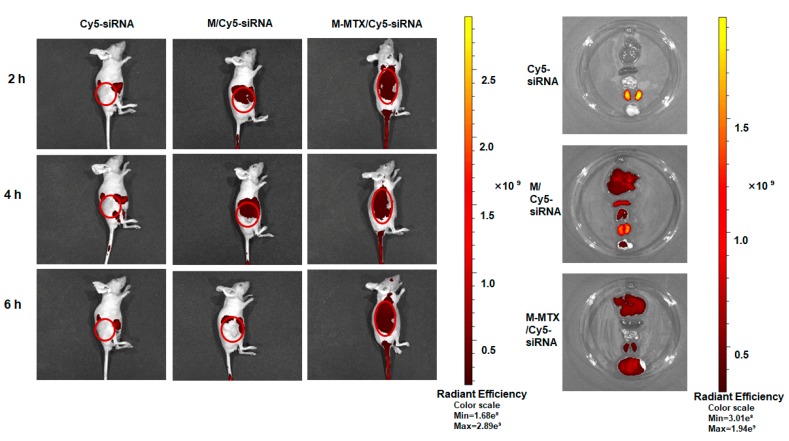
The biodistribution of M-MTX/Cy5-siRNA in tumor-bearing mice. (**Left**) The real-time fluorescence imaging of tumor-bearing mice injected with naked Cy5-siRNA, M/Cy5-siRNA, and M-MTX/Cy5-siRNA via tail vein were obtained at the second, fourth, and sixth hours after administration (Cy5-siRNA = 1 nmol). (**Right**) The ex vivo imaging of the tumors and organs excised from tumor-bearing mice at the sixth hour after injection. The images were obtained by IVIS® In Vivo Imaging System with an optimized parameter (excitation, 640 nm; emission, 680 nm).

The efficient delivery of siRNA and MTX to the targeting site was a prerequisite for a successful treatment. Therefore, we investigated the biodistribution of M-MTX/siRNA in tumor-bearing mice. Mice injected with M-MTX/Cy5-siRNA showed a higher accumulation (about 6-folds higher radiant efficiency) in solid tumors than the M/Cy5-siRNA-treated group without MTX conjugated (Figure 5). At the sixth hour, the red fluorescence signal in the tumor injected with Cy5-siRNA and M/ Cy5-siRNA was barely visible. The same results were also reflected in the anatomical organs (Heart, Liver, Spleen, and Lung). The fluorescence signal of the free Cy5-siRNA injection group was barely detectable in major organs and tumors other than the kidney. This indicated a fast clearance of free siRNA in vivo. However, M-MTX/Cy5-siRNA exhibited the lowest fluorescence intensity in the kidney. This suggested that the Cy5-siRNA loaded in M-MTX had a long circulation time. Compared to M-MTX/Cy5-siRNA, M/Cy5-siRNA had a higher fluorescence signal in the spleen, liver, lung, and kidney but had a lower tumor fluorescence signal. This may be due to the larger size, higher positive charge, and non-targeting ability of the M/Cy5-siRNA which was easily captured by the reticuloendothelial system. The high and specific accumulation of M-MTX/Cy5-siRNA in tumor tissues further indicated a potential and promising imaging application of the carrier in the clinic. 

### 3.9. Antitumor Efficacy of M-MTX/siRNA In Vivo

**Figure 6:**
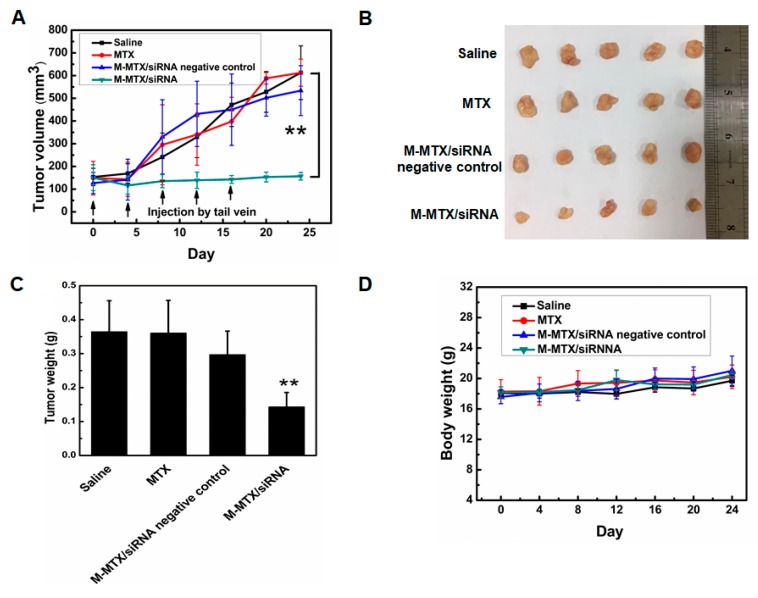
The therapeutic efficacy of M-MTX/siRNA in vivo. The tumor volume (**A**), photographs of the solid tumor (**B**), the tumor weight (**C**) of the animals treated with various drug formulations were obtained on day 24. (*n* = 5, ** *p* < 0.01, M-MTX/siRNA compared to saline). The body weight of the tumor-bearing mice treated with various drug formulations was also measured to assess the system toxicity (**D**). The values are the mean ± SEM, *n* = 5.

The synergistic antitumor efficacy of MTX and survivin siRNA was further assessed in tumor-bearing mice. Consistent with the in vitro results, tumor growth inhibition and disease remission were significantly achieved in the M-MTX/siRNA treated group with a low MTX and siRNA dose (** *p* < 0.01, *n* = 5) (Figure 6A–C). The M-MTX/siRNA-treated group had a large tumor volume and weight reduction (about 75% and 63%, respectively) compared to other groups. The HeLa cell lines are known for their high tumor growth rate. The Saline, MTX, and M-MTX/siRNA negative control groups showed a rapid increase in tumor size and weight (Figure 6A–C). Compared to MTX, M-MTX/siRNA negative control group had a lower tumor weight (Figure 6C). This suggested that the M-MTX/siRNA negative control had a more enhanced antitumor effect than free MTX, probably due to its extended circulation time (Figure 5) and an enhanced cellular uptake mediated by FR (Figure 2). The body weight of the mice was measured to evaluate the toxicity of the formulations. As shown in Figure 6D, the body weight of the different groups increased steadily, indicating that the formulations had no significant toxicity. 

### 3.10. Protein Expression, Immunohistochemistry, and Histopathological Analysis 

**Figure 7:**
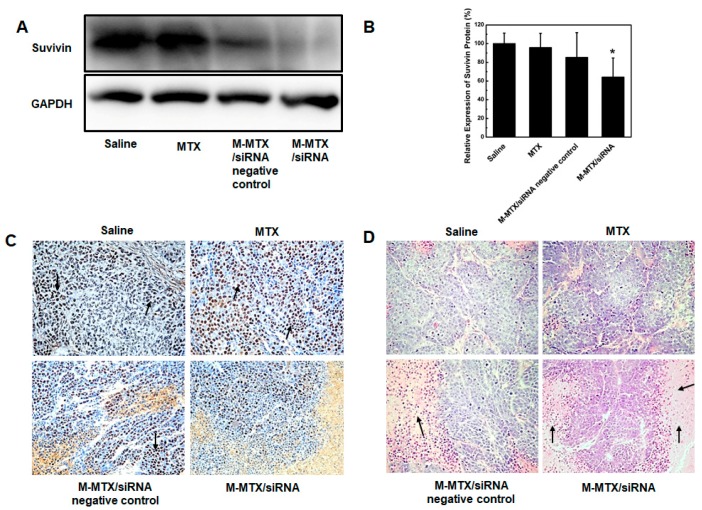
The protein expression, immunohistochemistry, and histopathological analysis of tumor tissues. Survivin expression in tumor tissue was determined by western blot assay (**A**) and then quantified by image J (**B**) (*n* = 3, * *p* < 0.05, M-MTX/siRNA compared to saline). The immunohistochemical analysis of protein expression (**C**) and the hematoxylin-eosin staining for tumor tissue necrosis (**D**) was also investigated.

**Figure 8:**
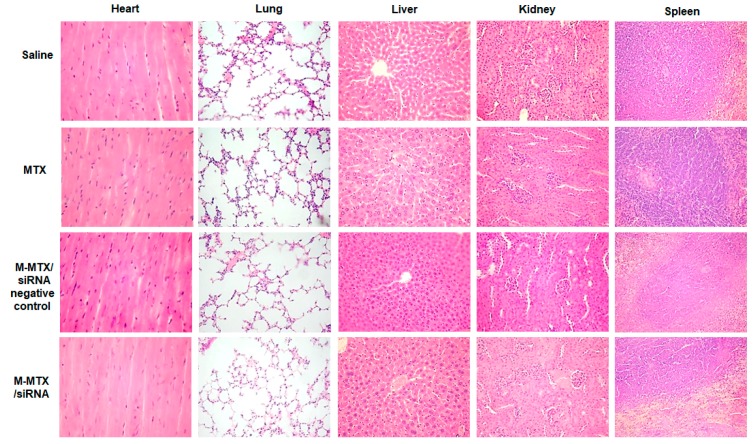
The organ toxicity analysis of different formulations: Major organs (Heart, Lung, Liver, Kidney, and Spleen) were dissected and stained with hematoxylin-eosin to assess the systemic toxicity of the drug formulations.

Survivin expression levels in tumors were also analyzed by a western blot and an immunohistochemical analysis (Figure 7A–C). The western blot results showed a 40% reduction in survivin expression in M-MTX/siRNA-treated tumors compared to the saline group (* *p* < 0.05, *n* = 3) (Figure 7A,B). The same results were also confirmed by the immunohistochemical analysis. The cells stained with brown and black nuclei were shown to be positive for survivin expression as indicated by the black arrow (Figure 7C). Furthermore, HE staining of the tumor tissues treated with M-MTX/siRNA showed a large amount of necrosis of the tumor cells as indicated by the black arrow (Figure 7D). The results of Figure 6 and Figure 7 showed that M-MTX could efficiently deliver siRNA to the cells in tumor tissue, suppressing the related expression of proteins and finally leading to the death of a large number of tumor cells in tumor tissues. There was no significant toxicity in the major organs of mice treated with MTX, M-MTX/siRNA negative control, or M-MTX/siRNA compared to the saline group due to the reduced dose of MTX and siRNA (Figure 8). M-MTX exhibited good biocompatibility and low toxicity as a non-viral delivery for siRNA delivery. 

## 4. Discussion

MTX, a FA analog, has long been used for cancer therapy but is often associated with severe systemic toxicity, bone marrow suppression, and drug resistance [6,7,43]. In addition, MTX monotherapy has limited effectiveness. Therefore, the new combination strategy of MTX and siRNA as well as a novel drug delivery system that reduces MTX toxicity and improves cancer efficacy is desirable [8,9]. As one of the strongest tumor apoptosis inhibitors, survivin not only promotes tumor cell proliferation but also is closely related to the development of tumor resistance [14,44,45]. Thus, the combination therapy of MTX and survivin-siRNA demonstrated a new possibility for enhanced tumor efficacy. In order to efficiently deliver MTX and survivin-siRNA into the cells, modified cationic polymer-based mixed micelles having an MTX targeting ability were designed due to their simplicity in synthesis and preparation.

LA is a polyunsaturated fatty acid that is essential for the body and is nontoxic. Moreover, recent studies reported that LA also inhibited tumor cell growth and metastasis [46,47,48]. Therefore, LA was selected to be separately conjugated to the b-PEI and mPEG to form two amphiphilic polymers of bPEI-LA and mPEG-LA in this work. The hydrophobic molecule of LA conjugated to bPEI was designed to reduce the high toxicity of bPEI [38]. MTX was then conjugated to the bPEI-LA by an esterase-stable amide linkage (Figure S1). MTX was conjugated to bPEI-LA by an amide linker to prevent and avoid MTX release in the blood and to enhance the targeting ability of MTX to the FA receptor. Then, M-MTX self-assembled by MTX-bPEI-LA and mPEG-LA was prepared and applied to efficiently co-deliver MTX and siRNA. M-MTX was aimed to target the tumor cells overexpressing FR and successfully release loadings into the cytoplasm as shown in Scheme 1. Actually, some researchers had revealed that MTX conjugated to the dendrimers showed a potential FR targeting ability through the tight-binding of MTX to the folate binding protein [49]. From the reversed cellular uptake (Figure 2) and biological activities (Figure 4) of M-MTX/Cy5-siRNA-treated groups preincubated with free FA and the high cellular uptake (Figure 2), biological activities (Figure 4) in vitro, and accumulation of M-MTX/Cy5-siRNA in the solid tumor (Figure 5), we point out that M-MTX nanocarrier exhibits an FR targeting ability and a much higher internalization efficiency via FA receptor-mediated endocytosis than M/siRNA complexes [50]. In Figure 4C, when M-MTX and MTX were incubated with cells for 4 h, the M-MTX-treated group exhibited a lower protein expression compared to free MTX. This also indicated that M-MTX entered more into the cells and had a higher internalization efficiency compared to free methotrexate. The siRNA and MTX-bPEI-LA conjugates with stable chemical linkage achieved endosome escape (Figure 3) and were released probably by the proton sponge effect of cationic polymer of bPEI [33,34]. As expected, M-MTX/survivin siRNA achieved tumor growth inhibition and disease remission with a low MTX and siRNA dose in tumor-bearing mice. There was no significant difference in the antitumor effect among saline, MTX, and the M-MTX/siRNA negative control with a low dose of MTX, which was not sufficient to achieve disease remission. However, the M-MTX/siRNA complexes exhibited an unexpectedly potent effect compared to MTX and the M-MTX/siRNA negative control. This may largely depend on the specific targeting and efficient delivery of siRNA by the bifunctional vector of M-MTX. Moreover, previous studies have shown that survivin siRNA may have a positive effect on chemotherapeutic drug sensitivity and may reduce drug resistance [14]. Thus, we propose that the effect of mutual promotion and the sensitivity of the two drugs at a low dose also contribute to the desired efficacy. The M-MTX carrier exhibited good biocompatibility and low toxicity as a non-viral carrier for siRNA co-delivery in vivo. However, the specific and detailed mechanisms and synergy of MTX and siRNA warrant further investigation.

## 5. Conclusions

In this study, an M-MTX/siRNA co-delivery system was developed based on the mixed micelles composed of mPEG-LA and MTX-bPEI-LA. M-MTX/siRNA exhibited a good ability to target tumor cells overexpressing FR. M-MTX/siRNA was internalized by FR-mediated endocytosis and was able to release siRNA into the cytoplasm. Studies in tumor-bearing mice showed that M-MTX/siRNA could greatly target tumor tissue and inhibit tumor growth in vivo. All above, M-MTX could be applied as a promising and safe nanoplatform for siRNA or even other therapeutic agent deliveries and may find utility in anticancer therapy.

## Supplementary Materials

The following are available online at https://www.mdpi.com/1999-4923/11/2/92/s1, Figure S1. The synthesis procedure of the amphiphilic polymers. mPEG-LA was synthesized by the reaction of oleic acid chloride and mPEG-NH2 (A). MTX-bPEI-LA was synthesized by two steps (B). BPEI-LA was first synthesized, and then, the MTX was conjugated to finally obtained MTX-bPEI-LA; Figure S2. The ^1^H NMR of mPEG-LA. mPEG-LA was dissolved in CDCl_3_ and measured in a nuclear magnetic resonance instrument (500 MH_Z_). The characteristic peaks of mPEG and LA were marked with the colour characters A, B, C, D, E, and F; Figure S3. The ^1^H NMR of bPEI-LA. bPEI-LA was dissolved in CDCl_3_ and measured in a nuclear magnetic resonance instrument (500 MH_Z_). The characteristic peaks of bPEI and LA were marked with the colour characters A, B, C, D, E, and F; Figure S4. The ^1^H NMR of MTX-bPEI-LA. MTX-bPEI-LA was dissolved in D_2_O and measured in a nuclear magnetic resonance instrument (500 MH_Z_). The characteristic peaks of bPEI, MTX, and LA were marked with the colour characters A, B, C, D, E, F, and G; Figure S5. The in vitro siRNA release profile of FAM-siRNA-loaded mixed micelles in PBS (pH = 7.4); Figure S6. The hemolytic analysis of M-MTX and MTX-bPEI-LA on murine erythrocytes (n = 3, mean ± SEM). 200 µL various concentrations of M-MTX and MTX-bPEI-LA were incubated with 1 mL erythrocytes in a 2% phosphate buffer saline for 3 h. The suspensions were centrifuged, and the absorbance of supernatant (100 µL) was measured at 450 nm; Figure S7. The cytotoxicity of M-MTX and MTX-bPEI-LA (*n* = 3, mean ± SEM). The cells were plated in 96-well microtiter plates (5000 cells per well) and cultured overnight. M-MTX and MTX-bPEI-LA at 3 concentrations (1, 5, and 20 µg/mL) were added. After another 24 h, the relative cell viability was determined and was presented as a percentage of the viability of untreated cells.
