# Supplementary material for: Targeted Co-Delivery of siRNA and Methotrexate for Tumor Therapy via Mixed Micelles
Source: Pharmaceutics. 2019 Feb 21;11(2):92. doi: 10.3390/pharmaceutics11020092 (PMC6409946; doi:10.3390/pharmaceutics11020092)
Supplement: Supplementary file 1 [file pharmaceutics-11-00092-s001.pdf]

pharmaceutics

Targeted Co-Delivery of siRNA and Methotrexate for Tumor Therapy via Mixed Micelles

Fei Hao, Robert J. Lee, Chunmiao Yang, Lihuang Zhong, Yating Sun, Shiyan Dong, Ziyuan Cheng, Lirong Teng, Qingfan Meng, Jiahui Lu, Jing Xie and Lesheng Teng

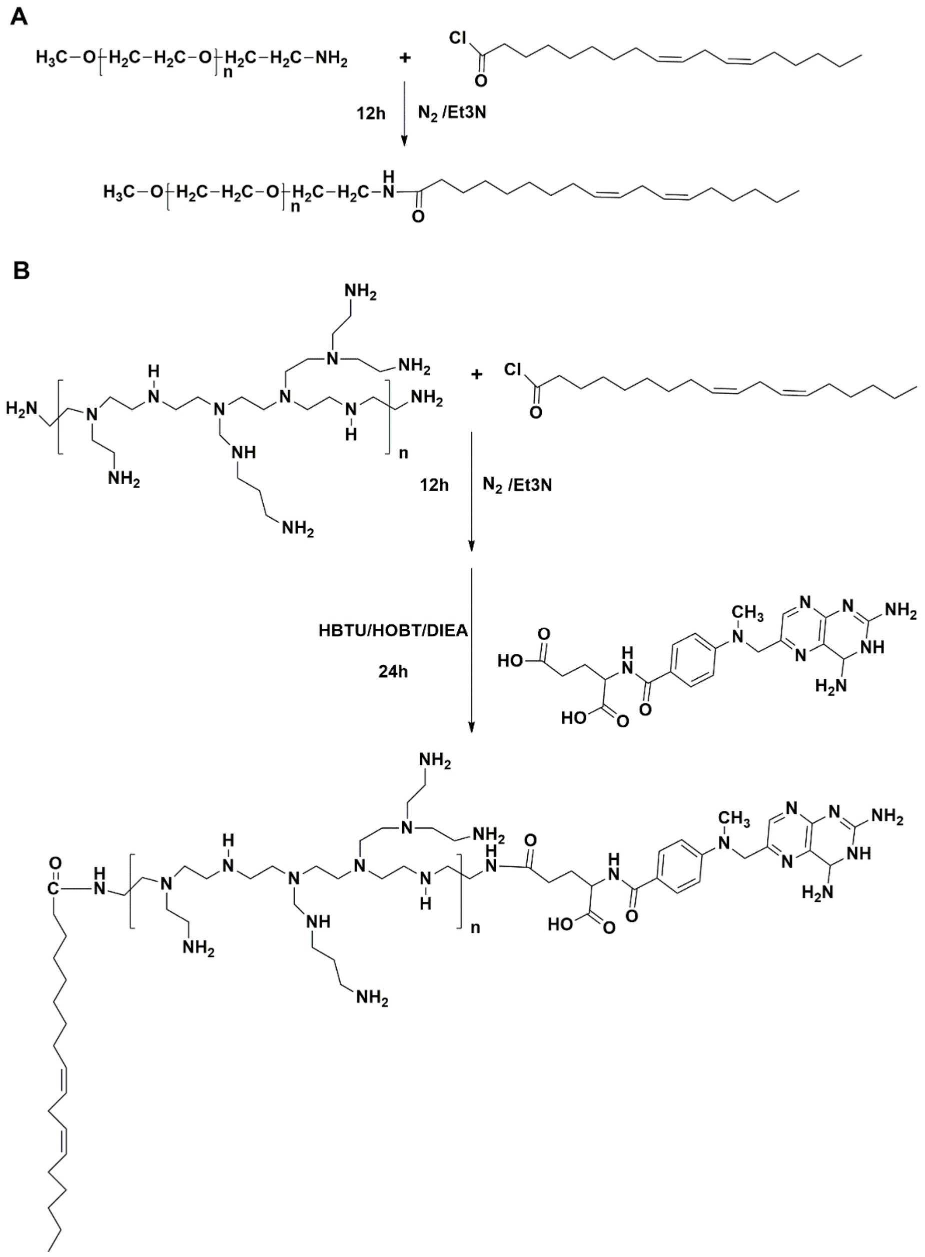

Figure S1. The synthesis procedure of the amphiphilic polymers. mPEG-LA was synthesized by the reaction of oleic acid chloride and mPEG-NH_2 (**A**). MTX-bPEI-LA was synthesized by two steps (**B**). BPEI-LA was first synthesized, and then, the MTX was conjugated to finally obtained MTX-bPEI-LA.

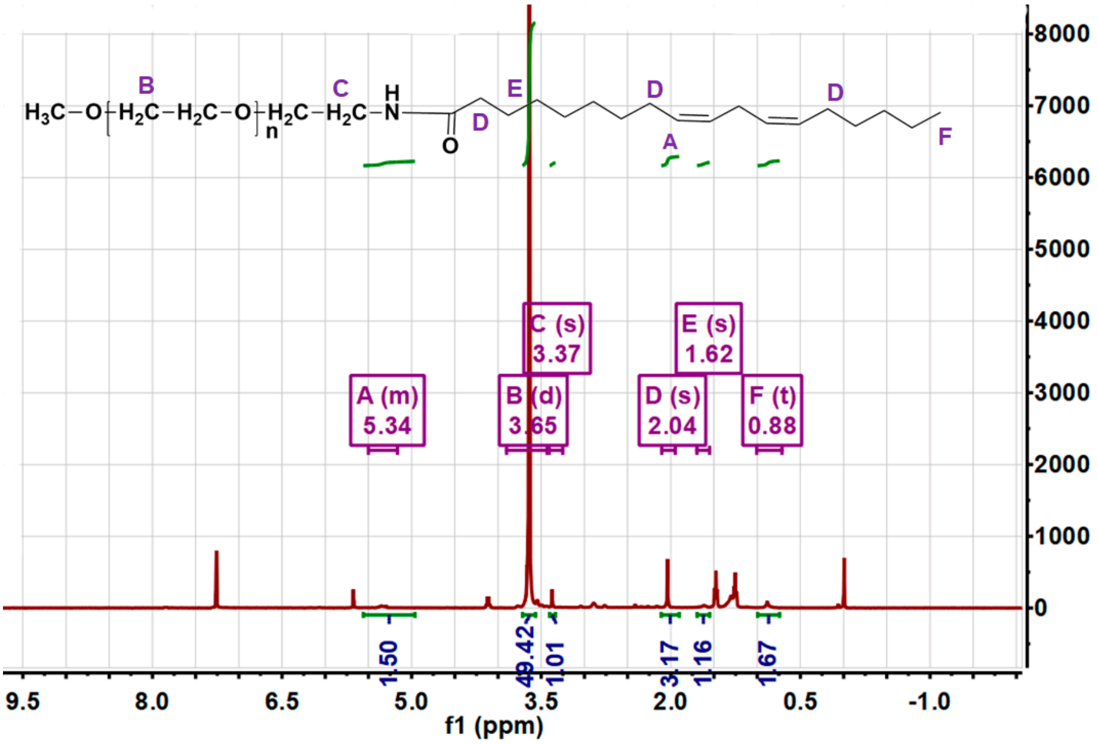

Figure S2. The ^{1}H NMR of mPEG-LA. mPEG-LA was dissolved in CDCl3 and measured in a nuclear magnetic resonance instrument (500 MHZ). The characteristic peaks of mPEG and LA were marked with the colour characters A, B, C, D, E, and F.

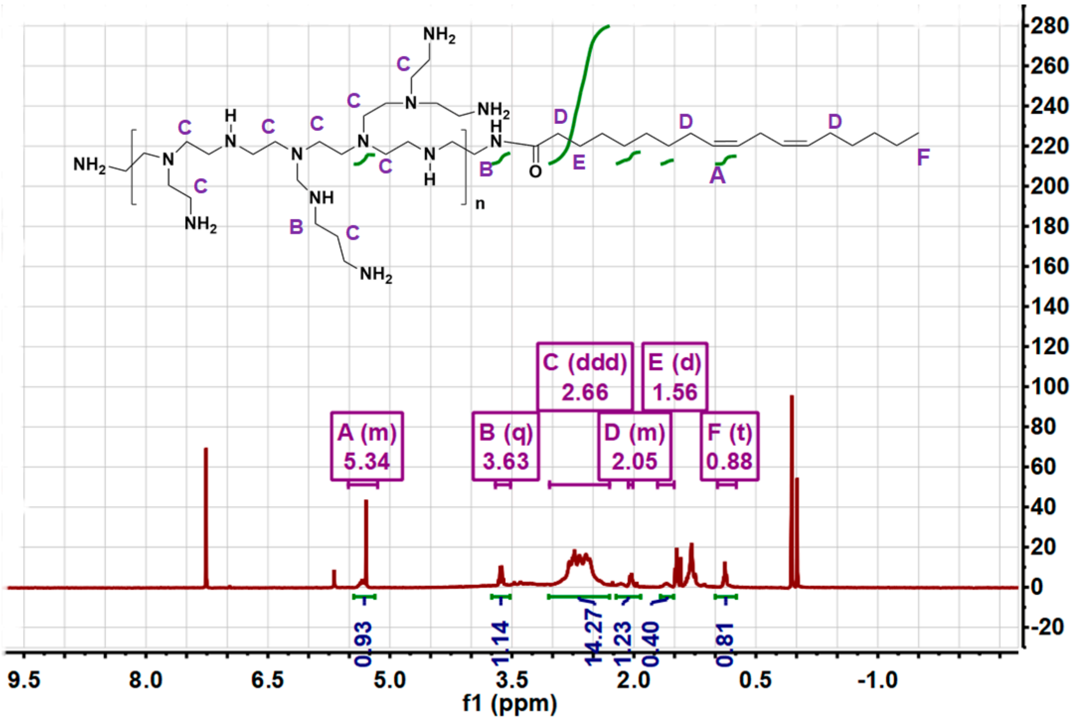

Figure S3. The ^{1}H NMR of bPEI-LA. bPEI-LA was dissolved in CDCl3 and measured in a nuclear magnetic resonance instrument (500 MHZ). The characteristic peaks of bPEI and LA were marked with the colour characters A, B, C, D, E, and F.

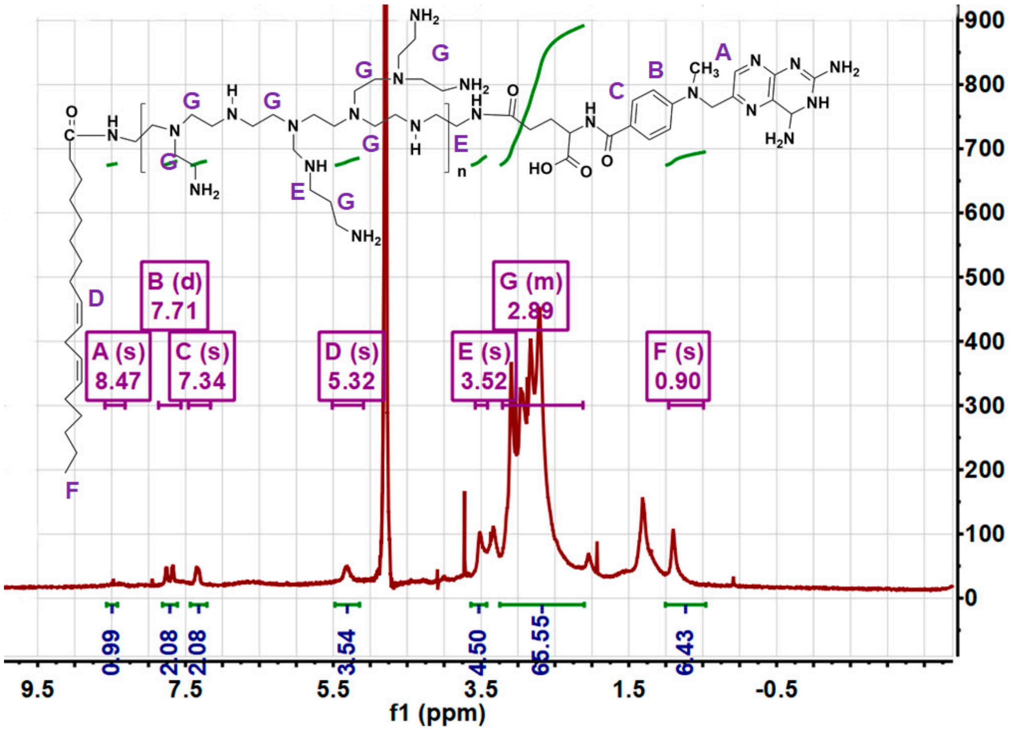

Figure S4. The ^{1}H NMR of MTX-bPEI-LA. MTX-bPEI-LA was dissolved in D_2O and measured in a nuclear magnetic resonance instrument (500 MHZ). The characteristic peaks of bPEI, MTX, and LA were marked with the colour characters A, B, C, D, E, F, and G.

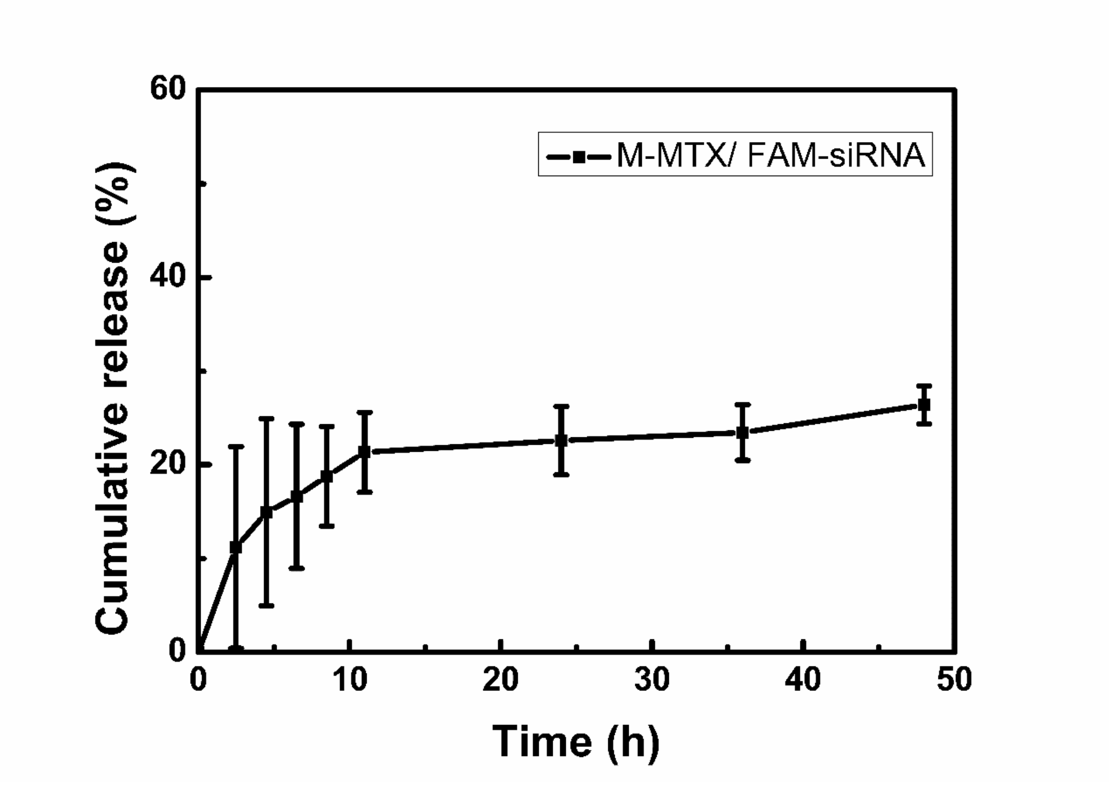

Figure S5. The in vitro siRNA release profile of FAM-siRNA-loaded mixed micelles in PBS (pH = 7.4).

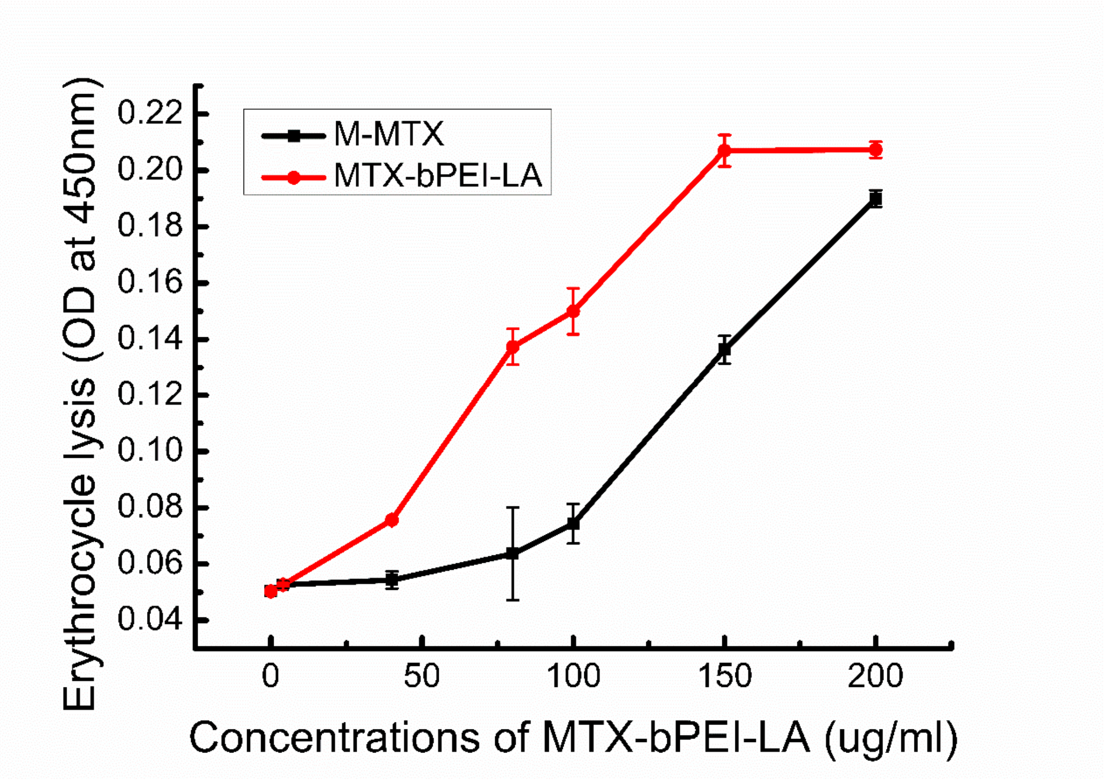

Figure S6. The hemolytic analysis of M-MTX and MTX-bPEI-LA on murine erythrocytes (n = 3, mean ± SEM): 200 μL various concentrations of M-MTX and MTX-bPEI-LA were incubated with 1 mL erythrocytes in a 2% phosphate buffer saline for 3 h. The suspensions were centrifuged, and the absorbance of supernatant (100 μL) was measured at 450 nm.

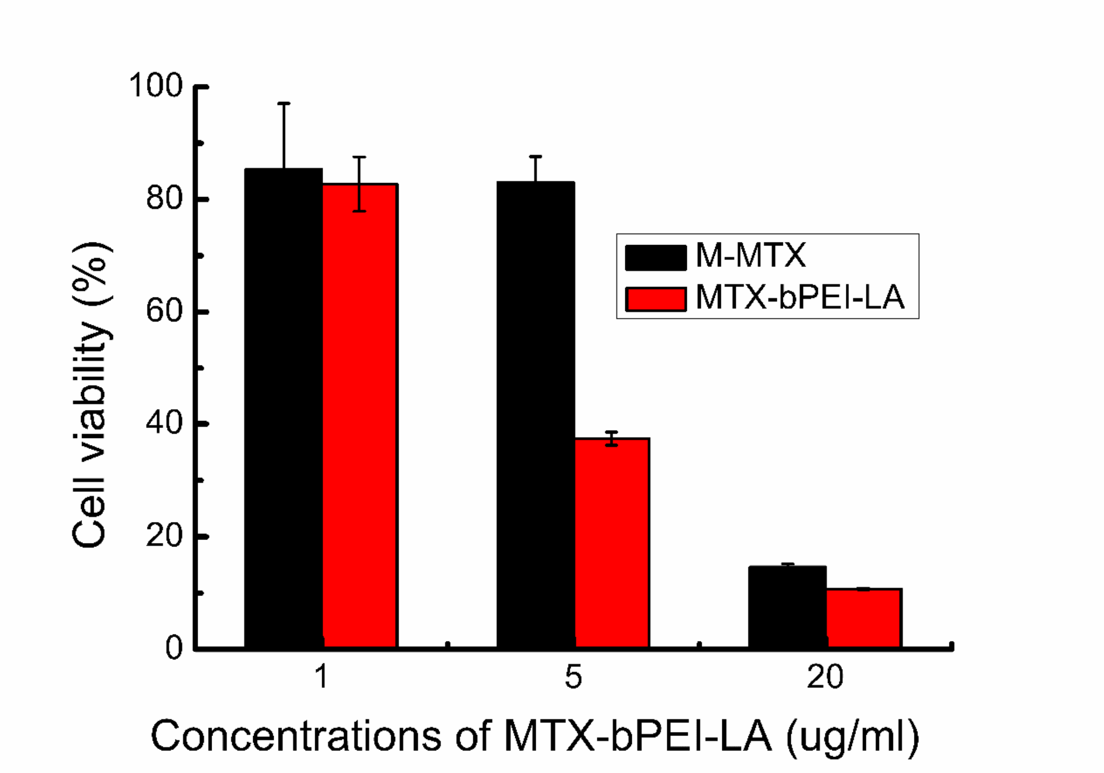

Figure S7. The cytotoxicity of M-MTX and MTX-bPEI-LA (n = 3, mean ± SEM). The cells were plated in 96-well microtiter plates (5000 cells per well) and cultured overnight. M-MTX and MTX-bPEI-LA at 3 concentrations (1, 5, and 20 μg/mL) were added. After another 24 h, the relative cell viability was determined and was presented as a percentage of the viability of untreated cells. .
